# General Practitioners and Pharmacists’ Perspectives on Electronic Prescribing for Multidose Drug Dispensing: Mixed Methods Study

**DOI:** 10.2196/85384

**Published:** 2026-05-06

**Authors:** Ann-Kristin Sørvik Rasmussen, Synne Mari Trælnes, Stian Skogly, Ole Kristian Hejlesen, Gunnar Hartvigsen

**Affiliations:** 1 Department of Computer Science, Faculty of Science and Technology, UiT The Arctic University of Norway, Hansine Hansens vei 54, Tromsø, 9037, Norway, 47 90657785; 2 Department of Research and Innovation, Helgeland Hospital Trust, Sandnessjøen, Norway

**Keywords:** electronic prescribing for multidose drug dispensing, eMDD, e-prescribing, e-prescriptions, multidose drug dispensing, medication safety, general practitioner, pharmacist, eHealth, health care communication, system usability, mixed methods

## Abstract

**Background:**

Medication safety remains a significant challenge in health care, particularly for patients managing complex treatment regimens. In Norway, the introduction of electronic prescribing (e-prescribing) for multidose drug dispensing (eMDD) aims to improve medication adherence and minimize errors by seamlessly integrating with the national e-prescription infrastructure.

**Objective:**

This study aimed to investigate the challenges faced by general practitioners (GPs) and pharmacists in using eMDD in Norway. Additionally, it sought to gather their recommendations for system improvements to guide future development and nationwide implementation.

**Methods:**

A parallel mixed methods design was used, integrating both quantitative and qualitative data. A structured online survey was distributed to 54 pharmacies and 190 GP surgeries across Norway. The survey included a combination of multiple-choice and open-ended questions. Qualitative responses were analyzed thematically using NVivo, while quantitative data were processed using the built-in analytical tools in Nettskjema.

**Results:**

A total of 70 health care professionals participated in the study, revealing 7 key themes: training, system and technology, communication and interaction, division of responsibilities, medication safety, time and resource use, and implementation challenges. GPs reported inadequate training and an overwhelming volume of communication, while pharmacists identified issues with system integration and unclear role definitions. Both groups emphasized the need for improved system usability, stronger interprofessional collaboration, and a more defined governance structure.

**Conclusions:**

While the eMDD system has the potential to improve medication safety and optimize workflows, its success depends on addressing technical inefficiencies, improving user training, and clarifying role responsibilities. Actively involving end users in system development and policy planning is critical for achieving effective national implementation and ensuring integration with broader eHealth initiatives, such as the Patient’s Medication List.

## Introduction

### Background

Medication safety remains a critical concern in health care, particularly for patients managing complex treatment regimens. In Norway, the multidose drug dispensing (MDD) system was introduced in the early 2000s to reduce manual errors and improve adherence by automating the packaging of medications into labeled sachets. Multidose refers to tablets and capsules for each dosing time that are mechanically packed into patient-specific bags on a continuous roll. Automated preparation, labeling, and pharmacist verification are intended to enhance adherence and minimize errors. The subsequent implementation of electronic prescribing (e-prescribing) for MDD (eMDD) has further improved adherence, increased accuracy in medication administration, and reduced the time burden for health care professionals [1].

Historically, multidose prescriptions were transmitted using paper-based methods, such as fax, PDF attachments, and postal mail. These processes introduced inefficiencies and increased the risk of medication errors due to inconsistent medication lists and delayed updates [1].

To address these challenges, Norway has developed and gradually implemented the eMDD system—as a further development of the multidose service. With eMDD, prescriptions are transmitted electronically through the national e-prescription infrastructure instead of papers or medication cards. This integration enables general practitioners (GPs) to send prescriptions via a centralized prescription intermediary, providing multidose pharmacies with real-time access to updated medication information. The system also supports the use of the Drugs in Use (*LIB: Legemidler i bruk*) message to ensure accurate and timely dispensing [2]. By replacing manual processes, eMDD aims to increase medication safety by ensuring timely medication delivery and supporting efficient workflows across health care services.

All municipalities have implemented electronic prescription systems; however, despite their potential to enhance medication safety and streamline workflows, the adoption of eMDD has been slow. By May 2025, out of 357 municipalities of the country, only 140 had implemented eMDD. Because a single municipality may encompass several general practices and pharmacies, municipal adoption does not fully reflect the system’s actual level of use. At the patient level, only about 21% of individuals receiving multidose medications are currently included in the e-prescribing solution, meaning that the remaining 79% still receive multidose rolls produced on the basis of paper-based prescriptions. This situation underscores the importance of establishing reliable and well-functioning electronic solutions now, as the coming years are expected to bring a substantial increase in conversions from paper-based to e-prescribing when the remaining patients transition into the system.

Barriers to adoption include the complexity of transitioning from paper-based systems, insufficient training, and limited interprofessional collaboration. Additionally, discrepancies in medication lists across care settings and unclear role responsibilities among health care providers continue to pose risks to medication safety [3].

The eMDD system is part of a broader national eHealth strategy, which includes initiatives such as e-prescriptions, the Summary Care Record, and the Patient’s Medication List. Together, these components aim to establish a unified digital infrastructure to support medication safety and continuity of care. Preliminary studies suggest that eMDD can improve communication, reduce errors, and enhance the quality of care. However, successful implementation requires more than technological readiness; it also demands organizational commitment, comprehensive user training, and well-defined governance structures [1,4]. Despite a systematic search in international databases, we found limited literature addressing eMDD outside Scandinavia. To our knowledge, the existing research is largely confined to Nordic health care systems, suggesting that eMDD may be a regionally specific phenomenon.

This study investigates the current state of eMDD implementation in Norway, identifies key facilitators and barriers, and explores its implications for medication safety and health care efficiency. The findings aim to inform strategies for scaling eMDD nationally and integrating it effectively within the broader eHealth ecosystem.

### Objective

The aim of this study is to investigate the challenges associated with the use of eMDD in Norway, as experienced by GPs and pharmacists. As the primary users of the eMDD system, these 2 professional groups offer valuable insights into its practical implementation and operational hurdles. Additionally, the study seeks to gather their recommendations for system improvements, with the ultimate goal of informing future development and facilitating the national scaling of eMDD. The central research question guiding this study is as follows: what are the challenges of using eMDD and how do GPs and pharmacists propose that these challenges be addressed?

## Methods

### Study Design

In order to answer the research question, we used a parallel mixed methods design, combining quantitative and qualitative data. Mixed methods in a research context consist of different design categories: explanatory, exploratory, parallel, and embedded. We chose the parallel design to gain both measurable results and a deeper understanding of participants’ perspectives, feelings, and motivations in addition to quantitative data. Although slightly unconventional, this approach was selected to capture how users experience and interact with eMDD.

In this study, our research group chose to focus exclusively on participants who had already begun using eMDD. This approach was motivated by the need to obtain authentic feedback on challenges and perhaps, most importantly, suggestions for improvement from pharmacists and physicians with practical experiences. Including respondents who had not yet adopted eMDD could have resulted in speculative answers that lacked real insight into the user experience and might have even provided a misleading basis for system enhancements.

To avoid this, we obtained information from all major pharmacy chains and the Norwegian Health Network to identify which pharmacies and medical practices had implemented eMDD. This allowed us to target the survey exclusively to active users of the service.

Regarding the study design, interviews could have provided more in-depth responses. However, given the large pool of potential respondents, this would have been too resource-intensive within the available timeframe. Each pharmacy or medical practice receiving the email could potentially have provided multiple responses, and each municipality includes several such sites. For this reason, we opted for a questionnaire-based approach, which enabled us to reach a broad audience and collect a substantial amount of valuable data. To ensure a practical and efficient approach, we opted for a single, unified questionnaire rather than separate surveys for each professional group. This decision was motivated by the need to reduce complexity and encourage participation, as longer or multiple surveys could have discouraged responses. A shared instrument also provided a stronger basis for comparing similarities and differences between GPs and pharmacists. Although the survey was anonymous, respondents indicated their professional role, allowing us to differentiate responses during the analysis. Furthermore, using identical questions enabled us to identify challenges and improvement suggestions relevant across systems and professions, while maintaining a comprehensive perspective. For future research—or in a repeated survey after further system development—it could be valuable to explore each professional group in greater depth.

Considering the busy work schedules of both physicians and pharmacists, a survey that could be completed at any time was more practical than arranging joint interview sessions.

Once eMDD is adopted, there is virtually no return to previous practices. We have not encountered any cases of reverting to paper-based systems after transitioning to eMDD, although this observation is anecdotal and not formally documented. For medical practices, the adoption is binary—they either use eMDD or they do not. Pharmacies, however, may receive eMDD prescriptions from 1 medical practice while still handling paper-based multidose prescriptions from another. Consequently, the number of patients converted to eMDD can vary significantly between pharmacies.

### Data Collection

A structured questionnaire was developed and distributed using Nettskjema, a secure and user-friendly online survey platform (Multimedia Appendix 1) [5]. The questionnaire included a combination of single-choice, multiple-choice, matrix, and open-ended questions, with a primary emphasis on qualitative responses to capture user experiences. The survey was designed to be anonymous, concise, and accessible, with an estimated completion time of 10 to 15 minutes.

To ensure clarity and relevance, the questionnaire was piloted with 6 health care professionals experienced in eMDD, including GPs, pharmacists, and a municipal pharmacist. Feedback from the pilot study was used to refine the final version of the questionnaire.

The survey was distributed via email to 54 pharmacies and 190 GP surgeries across Norway. Efforts were made to maximize the number of participants, including follow-up attempts, to address undeliverable emails. The survey remained open for 14 days, with a reminder sent after 8 days.

### Ethical Considerations

Ethical approval for the study was obtained from Sikt (ref. no. 887722), the Norwegian Agency for Shared Services in Education and Research [6]. Participation was entirely voluntary and anonymous, with informed consent obtained through the survey introduction. No compensation was provided to participants. All data were handled in accordance with the privacy regulations reviewed by Sikt and securely stored.

### Data Analysis

Quantitative data were analyzed using the built-in tools available in Nettskjema. For qualitative data, a thematic analysis was conducted following the 6-step process outlined by Braun and Clarke [7]. The process included (1) familiarization with the data, (2) generating initial codes, (3) developing themes, (4) reviewing themes, (5) defining and naming themes, and (6) reporting the findings.

**Figure 1.**
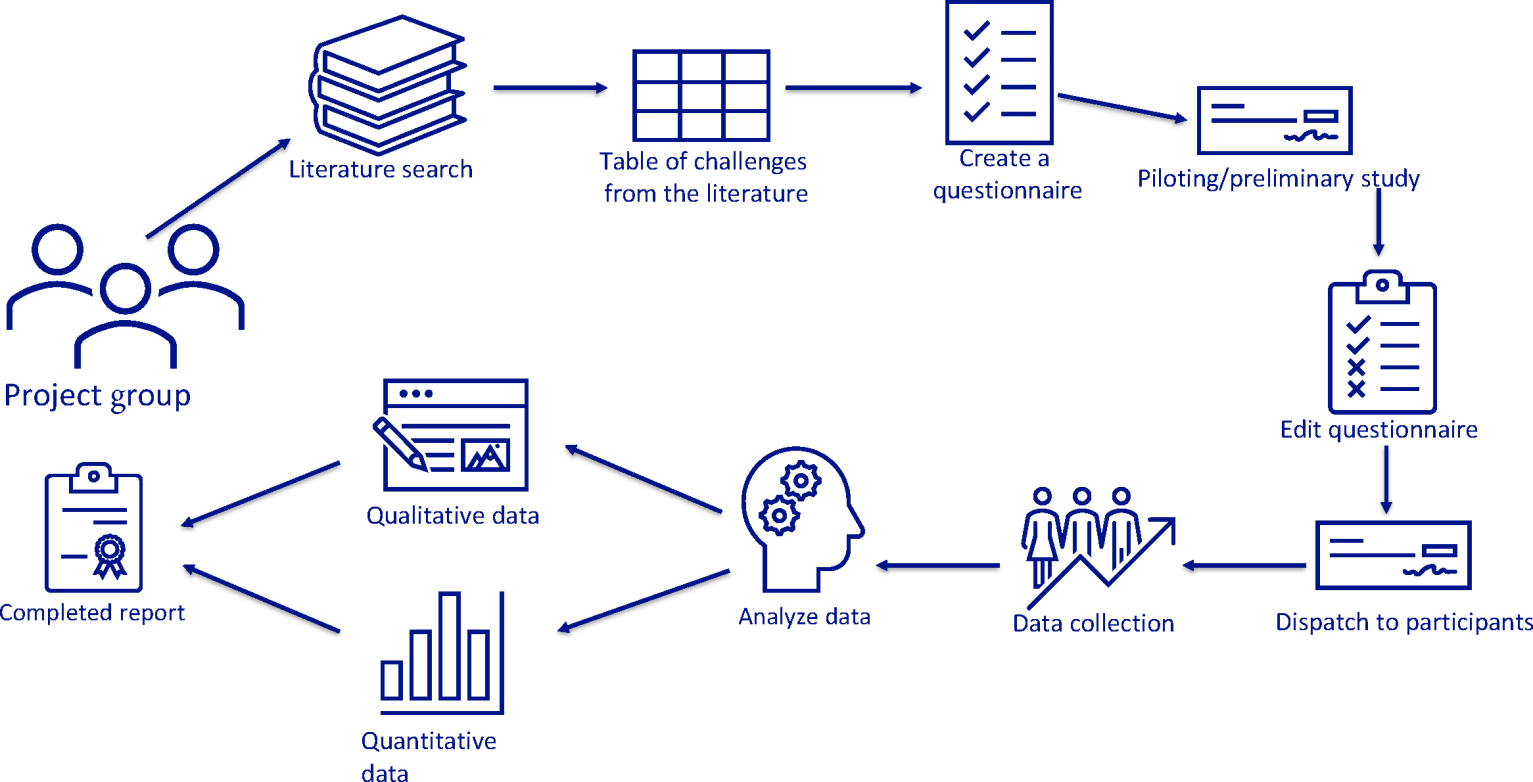
Illustration of the study’s methodological process from start to finish.

The analysis was supported by NVivo (Lumivero), a qualitative data analysis software approved for use by the University of Tromsø. NVivo facilitated systematic coding and categorization of responses, enabling meaningful comparisons between the 2 professional groups. Figure 1 provides an overview of the study’s methodology, detailing each step from start to finish.

## Results

### Participant Overview

A total of 70 health care professionals, including GPs (n=55) and pharmacists (n=15) with experience using eMDD, participated in the survey. Thematic analysis of their responses revealed 7 overarching themes: challenges with eMDD, training, system and technology, communication and interaction, division of responsibilities, medication safety, and time and resource use.

The survey’s first question used a multiple-choice table to map participants’ experiences with challenges and to identify any additional issues. Each main topic was explored through 2 lenses: current challenges and potential improvements. The results are presented by topic and categorized by professional groups (GPs and pharmacists), highlighting key differences in their experiences and perspectives. All participant quotes in this paper were translated from Norwegian into English using Copilot (Microsoft).

### Challenges With eMDD

The survey question for eMDD was as follows:

*How often do you experience challenges around these areas in your work with e-prescribing for multidose drug dispensing?*

**Figure 2.**
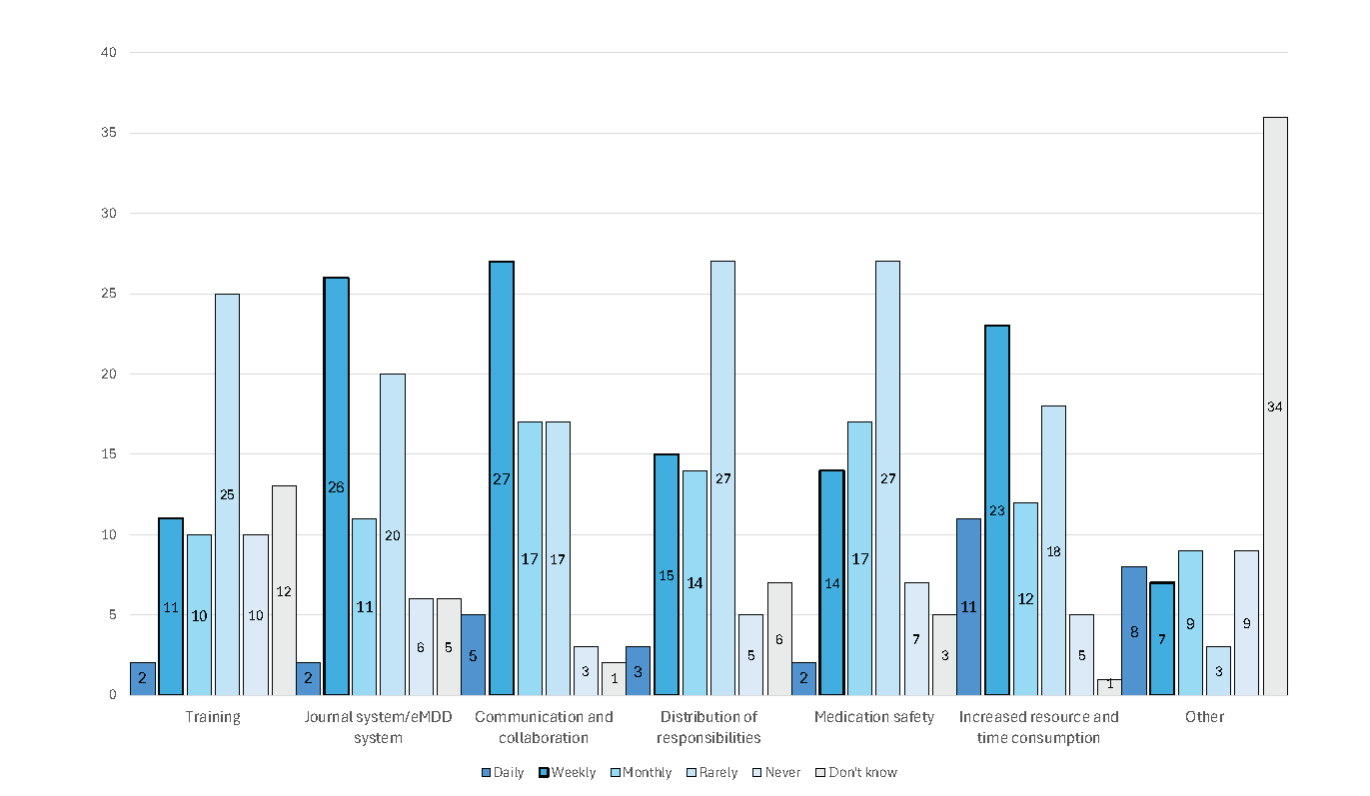
Participants’ responses on how often they experience challenges with electronic prescribing for multidose drug dispensing (eMDD).

Participants reported varying frequencies of challenges in their daily work with eMDD. The most commonly mentioned issues included system-related problems, communication barriers, and time and resource constraints. While some participants indicated that challenges were infrequent, others reported experiencing disruptions on a daily or weekly basis. Figure 2 illustrates these responses.

Notably, distinct differences emerged between the 2 professional groups:

- Pharmacists more frequently reported issues related to system integration and technical functionality.
- GPs highlighted challenges associated with communication overload, particularly in coordinating with other health care professionals.

These findings underscore the distinct perspectives of each group, emphasizing the need for tailored solutions to address their specific challenges.

### Training

#### Reported Challenges

The survey question for training was as follows:

*How has training in the use of e-prescribing for multidose drug dispensing been for you in your workplace?*

**Figure 3.**
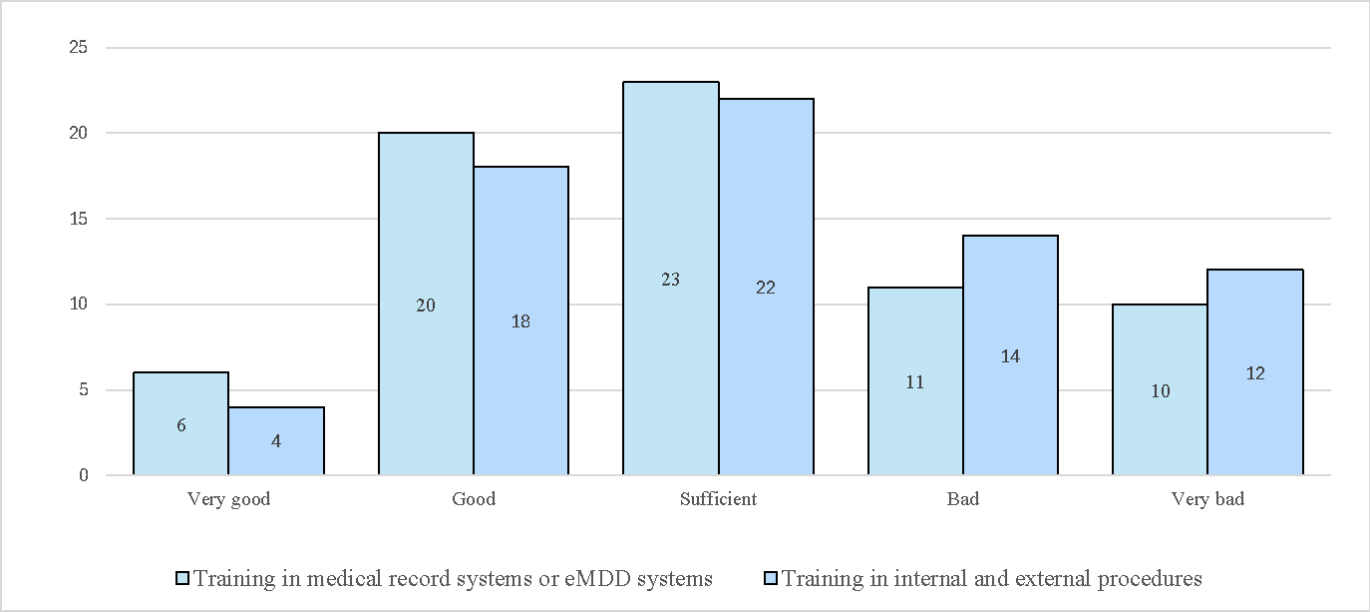
Responses from participants about their experiences of training in the use of electronic prescribing for multidose drug dispensing (eMDD).

Survey responses revealed mixed experiences regarding training in the use of eMDD. While many participants rated their training as “good” or “sufficient,” others described it as “poor” or “very poor.” There is a notable difference between the 2 professional groups: pharmacists generally rated their training experiences positively, whereas GPs were more likely to rate their training experiences negatively. Figure 3 illustrates these responses.

GPs highlighted the need for improved access to educational resources, such as e-learning modules, instructional videos, websites, or written materials. They also recommended implementing periodic reviews of training after some time using eMDD, as well as mandatory training for all users. Additionally, GPs called for improved user support and continuous follow-up from the system supplier to address challenges effectively.

#### Quotes From GPs

*Lack of training is the main problem. If there is a short intro on a website about e-prescribing for MDD, if not—there is a clear need.*

[GP]

*The system freezes, and the feedback has its own tribal language. There are many reports to and from before a multidose is established.*

[GP]

Pharmacists suggested clearer training plans and more practical, hands-on training. They also emphasized the importance of collaborative training with GPs to foster better communication and understanding of each other’s workflows. This, they suggested, could help reduce waiting times and improve overall efficiency.

#### Quotes From Pharmacists

*Took some time in the beginning before the GP cracked the code on how this worked. It is desirable that the GPs also receive training in this, and that this should not be self-taught. They are probably busy enough already without having to learn this themselves.*

[Pharmacist]

*I would like to have a better overview of the system from the GP’s side so that we can understand challenges and possibly help solve them.*

[Pharmacist]

Participants from both groups identified several areas for improvement in training:

- The need for more training time and ongoing support
- The importance of user-friendly, intuitive systems in reducing the learning curve
- Enhance collaboration between pharmacists and GPs to improve mutual understanding and streamline processes

These findings highlight the importance of tailored, comprehensive training programs that address the challenges faced by each professional group while fostering better interdisciplinary collaboration.

### System and Technology

#### Reported Challenges

The survey question for system and technology was as follows:

*What challenges have you experienced with the e-prescribing for multidose drug dispensing you use in your workplace?*

**Figure 4.**
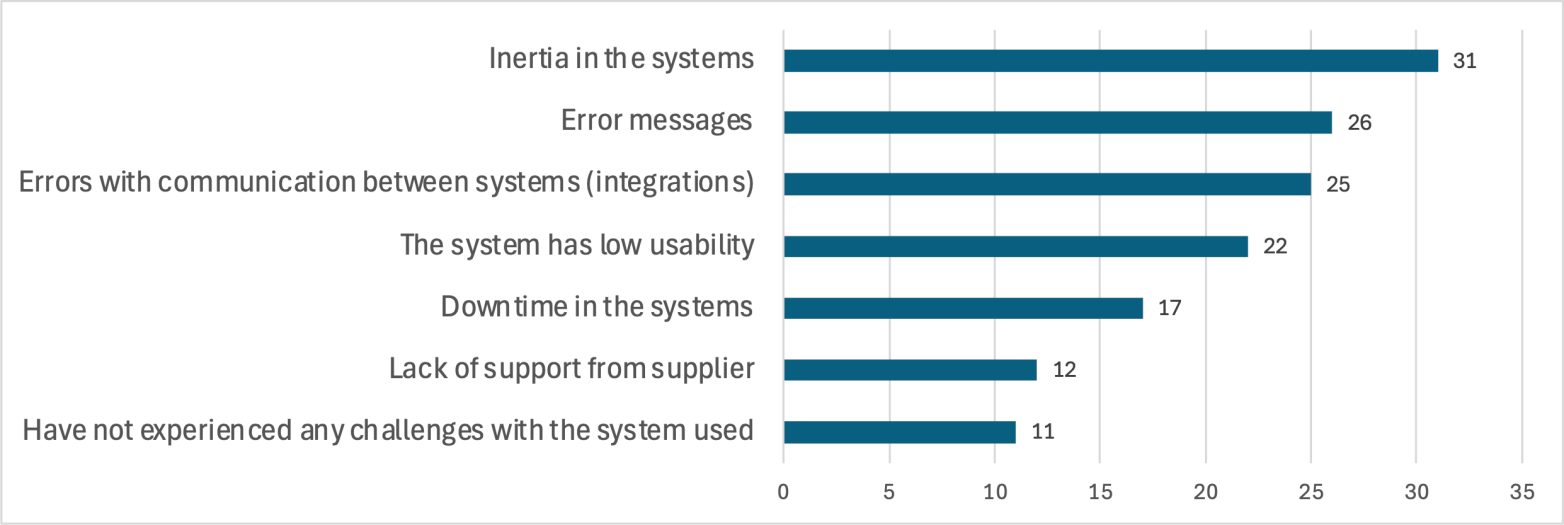
Participants’ responses to the challenges they experience with systems or the technology used in their work with electronic prescribing for multidose drug dispensing.

The majority of participants reported various challenges with the eMDD system, including sluggish performance, integration errors, frequent error messages, and low usability. Notably, only 11 out of 70 participants indicated that they did not experience any issues. GPs mainly reported concerns related to system slowness, error messages, and poor user-friendliness, whereas pharmacists highlighted issues such as system inertia, downtime, and integration errors. Figure 4 illustrates these responses.

Participants described the eMDD systems as lacking user-friendliness and suggested several improvements, including enhancing usability and simplifying processes, improving functionality to reduce errors and minimize unnecessary tasks, and introducing new features to address recurring challenges. Specific concerns were raised about the duplication of work, particularly when patients are hospitalized or have private multidose arrangements, with hopes that Central Prescribing Module and Patient’s Medication List solutions could address these issues. Additionally, technical problems and system slowness were noted as causing time loss and work interruptions. Unnecessary requests and unclear system changes were also identified as key weaknesses that need to be addressed.

#### Quotes From GPs

*Less rigid in terms of generic substitution, dosage form, length of prescriptions and not having to renew prescriptions that are in multidose.*

[GP]

*A lot of notifications, the system sends too many automated messages. I have experienced that they send a message for renewal of prescriptions, even though there is a lot left on the prescription.*

[GP]

#### Quotes From Pharmacists

*I want a more user-friendly system and a more advanced system that means that the risk of errors is minimal.*

[Pharmacist]

*A dialogue message about missing a prescription should have gone directly to a GP, and it should have been possible to send a dialogue message to the hospital physician regarding prescriptions reimbursed by hospitals.*

[Pharmacist]

### Communication and Interaction

#### Reported Challenges

The survey question for communication and interaction was as follows:

*What do you find challenging about communication and interaction between the health services?*

Challenges related to communication and interaction were commonly reported by eMDD users. Key issues included an increased number of dialogue messages, misunderstandings between services, and a lack of standardization in routines and systems. However, 13 out of 70 participants indicated that they did not experience these challenges.

**Figure 5.**
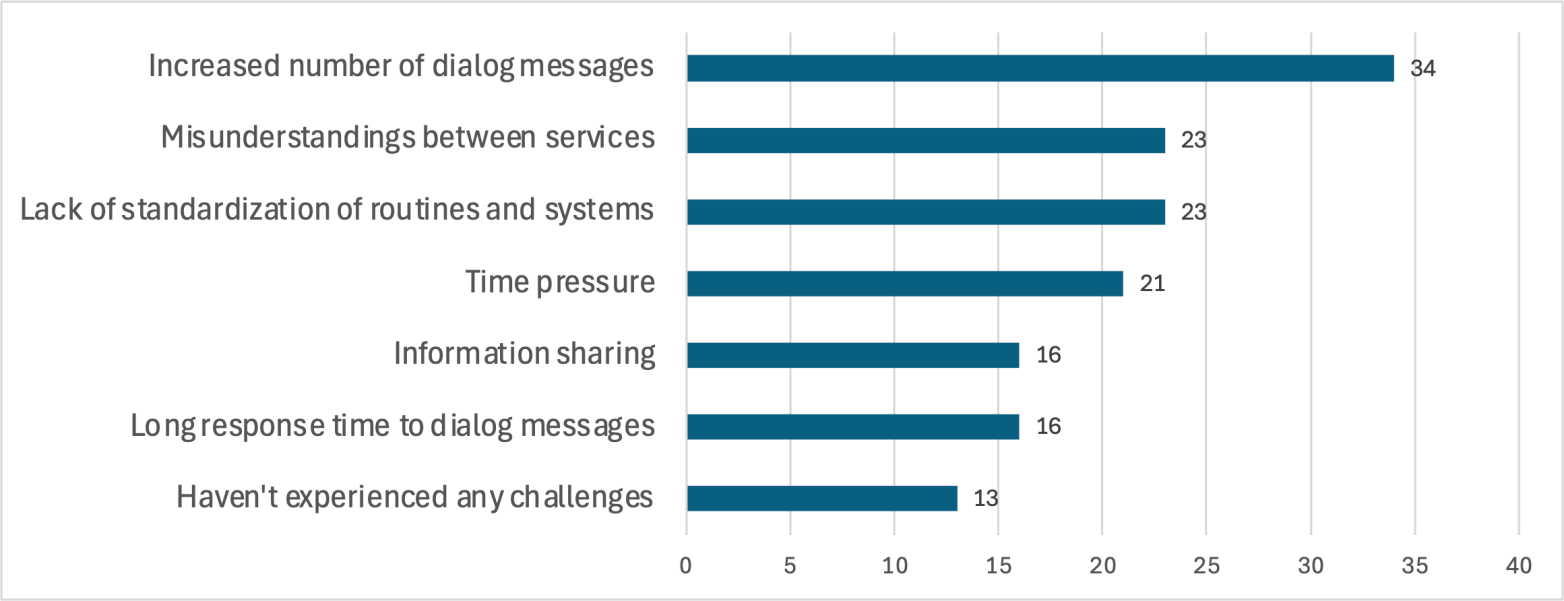
Participants’ responses about challenges they face with systems and technology used in electronic prescribing for multidose drug dispensing workflow.

GPs mainly reported an increased number of dialogue messages and misunderstandings between services, whereas pharmacists highlighted long response times and time pressure as significant challenges. Figure 5 illustrates these responses.

GPs recommended a clearer division of responsibilities for medication changes, better documentation in discharge summaries, improved information flow, and fewer but more relevant dialogue messages, as responding to excessive messages was described as time-consuming.

#### Quotes From GPs

*Not sending so many messages from the system to the GPs. Enough with one message about one case, not 10 every day!*

[GP]

*There should be a common communication platform for pharmacies, GPs and home care services.*

[GP]

Pharmacists emphasized the need for clearer prioritization of dialogue messages and prescription requests, as well as faster handling of requests to avoid delays in multidose packaging.

#### Quotes From Pharmacists

*The need for renewal of prescriptions should go directly to a GP, so that the pharmacy does not have to ask for every time it is empty.*

[Pharmacist]

*We get prescription messages in the system for some e-prescriptions that are not in the prescription intermediary, and the GP is unable to remove or enter new valid prescriptions.*

[Pharmacist]

Some participants proposed developing a shared communication platform for pharmacies, GPs, and home care services. Such a platform could streamline communication, reduce misunderstandings, and improve overall efficiency. Additionally, participants noted that technical issues often affect communication between parties, further emphasizing the need for improvements of the system.

### Division of Responsibilities

#### Reported Challenges

The survey question for division of responsibilities was as follows:

*What do you think has been challenging about the division of responsibilities related to e-prescribing for multidose drug dispensing?*

**Figure 6.**
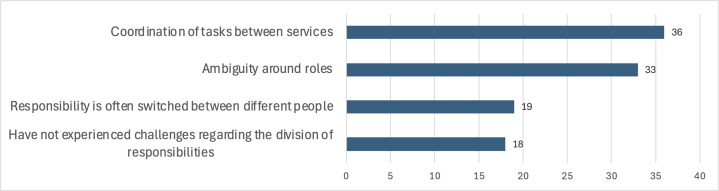
Challenges participants have experienced related to the division of responsibilities.

The division of responsibilities within the eMDD workflow was identified as a significant challenge, particularly in terms of task coordination and role ambiguity. Moreover, 19 out of 70 participants reported frequent shifts in responsibility between individuals, whereas 18 participants did not experience such challenges. Figure 6 illustrates these responses.

GPs primarily noted issues with task coordination, although some reported no challenges with responsibility division. In contrast, nearly all pharmacists highlighted role ambiguity and frequent shifts in responsibility as key concerns.

Some participants expressed uncertainty about the roles and responsibilities within the eMDD pathway, particularly regarding hospital doctors. They suggested a clearer definition of responsibilities between municipalities and hospitals, as well as improved internal routines to clarify roles.

Participants noted that responsibilities often change hands, leading to forgotten tasks and issues that only the patient’s primary GP could address. This problem was particularly evident during holidays or when temporary workers were involved, causing delays in prescription renewals or necessary adjustments.

Challenges with task coordination were especially evident when patients were admitted to hospitals or changed GPs, leading to disruptions in the eMDD workflow and further complicating the process.

#### Quotes From GPs

*Clear internal routines in the different locations. Maybe also useful with some more communication at a higher level [in management].*

[GP]

*We have had several different substitute GPs who have to register as a GP in charge of multidose when they are here. High risk of things slipping.*

[GP]

#### Quotes From Pharmacists

*All functions should be familiar with their roles and responsibilities, so that the pharmacy does not interfere in prescription orders or dose changes that the pharmacy does not have an overview of, or that the multidose is not stopped because a prescription was not renewed.*

[Pharmacist]

*When the patient switches to a GP who is affiliated with e-prescribing for MDD, but the pharmacy has not been notified.*

[Pharmacist]

### Medication Safety

#### Reported Challenges

The survey question for medication safety was as follows:

*What do you think is challenging about medication safety related to e-prescribing for multidose drug dispensing?*

**Figure 7.**
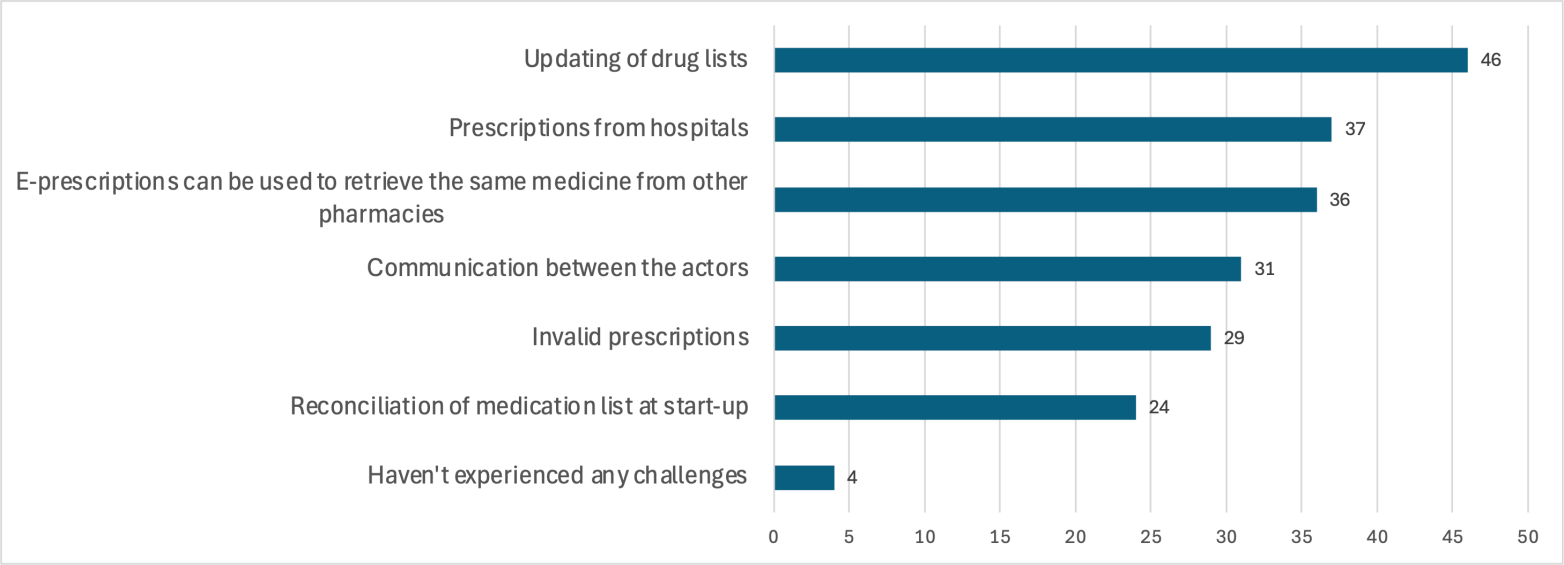
Challenges participants have experienced related to medication safety.

Medication safety was the survey’s most frequently discussed theme. Participants identified several key challenges, including updating medication lists, managing hospital prescriptions, and ensuring effective cross-functional communication. Updating medication lists was highlighted by 46 out of 70 participants, mostly GPs. Additionally, 36 participants noted that e-prescriptions used in multidose pharmacies can also be used at other pharmacies, creating risks of duplicate dispensing. This concern was particularly significant for A/B drugs. To address this, participants suggested locking e-prescriptions to the patient’s multidose setup and ensuring that other pharmacies are informed about the eMDD enrollment. Pharmacists mainly reported issues with invalid prescriptions and reconciling medication lists for new eMDD. Notably, only 2 GPs and 2 pharmacists reported no challenges with medication safety. Figure 7 illustrates these responses.

GPs and pharmacists suggest establishing clearer *routines* and better interaction when updating medication lists or initiating eMDD. One participant proposed implementing automatic notifications to inform all relevant parties about changes to medication lists.

#### Quotes From GP

*Implement better notification systems that automatically notify all actors involved in the event of medication changes.*

[GP]

*It must be possible to lock e-prescribed multidose drugs so that they cannot be duplicated and/or in large quantities by the patient at other pharmacies.*

[GP]

#### Quotes From Pharmacists

*Reconciliation of medication list for patients at the start of e-prescribing for MDD. I find that many GPs transfer their patients to e-prescribing for MDD in connection with changes, which makes it difficult for the pharmacy to agree or decide whether any medication is missing or discontinued.*

[Pharmacist]

*Possibility to lock MDD e-prescriptions so that they cannot be collected at other pharmacies by mistake.*

[Pharmacist]

### Time and Resource Use

#### Reported Challenges

The survey question for time and resource use was as follows:

*How has the use of e-prescribing for multidose drug dispensing led to increased time and resource use in your workplace?*

**Figure 8.**
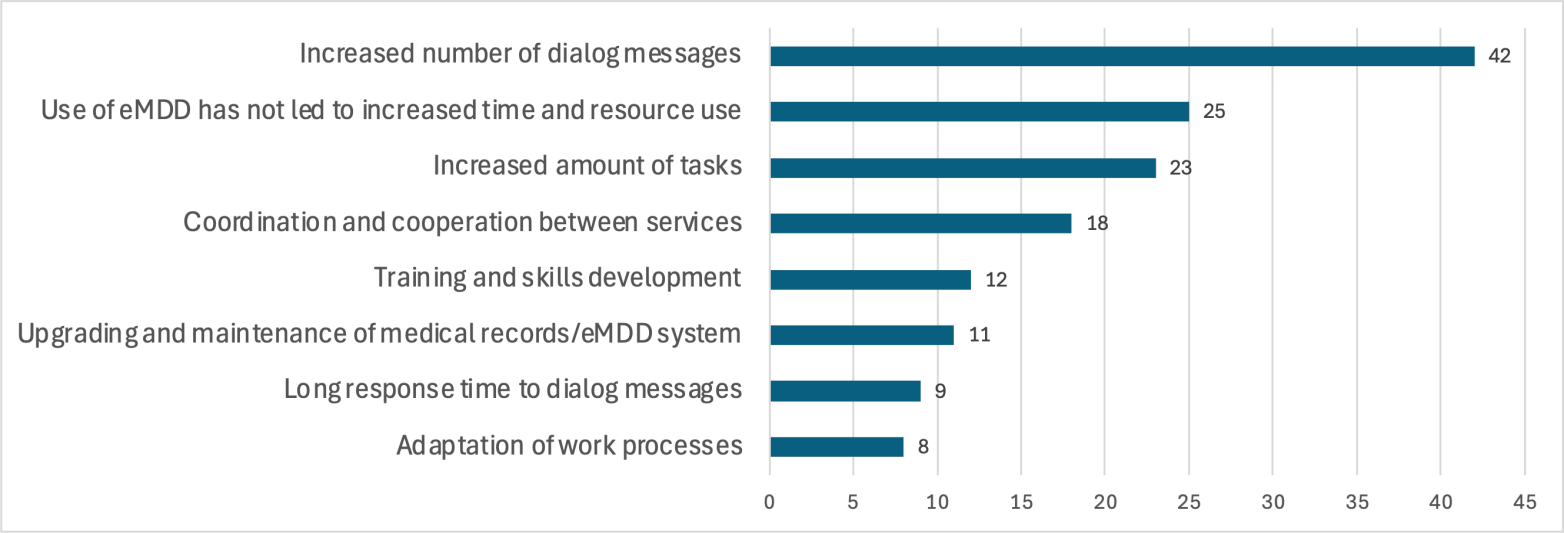
Reasons for increased time and resource use related to work with electronic prescribing for multidose drug dispensing (eMDD).

Participants identified increased tasks, dialogue messages, and coordination between services as the main challenges associated with eMDD. Many GPs noted that the rise in dialogue messages has contributed to increased time and resource demands, whereas pharmacists highlighted additional tasks and prolonged response times. However, 25 out of 70 participants, mostly pharmacists, stated that they did not experience increased time or resource use due to eMDD. Figure 8 illustrates these responses.

To address these challenges, participants suggested renewing multiple prescriptions simultaneously to reduce message exchanges. They also proposed enabling pharmacies to send eMDD messages directly to home care services to streamline communication. Additional recommendations included introducing more efficient eMDD features, such as notifications for important changes, the ability to view change reports, and a function to label multiple drugs for control or discontinuation. A better overview of messages was also highlighted as a priority.

Additionally, participants recommended developing a common communication platform for all stakeholders to reduce time and resource use. They also emphasized the importance of training and expressed optimism that the implementation of the Patient’s Medication List could help alleviate some of the additional workload.

#### Quotes From GPs

*It is absolutely necessary to review the regulations. A bit surprising that we get yet another request here when everything that is written has been carefully described before.*

[GP]

*Paying far too little attention to the GPs' busy everyday lives: meaningless messages from pharmacies about one request for renewal per prescription – when the patient has 20 medications, it MUST be possible to renew all of them in one process!*

[GP]

*It is absolutely necessary to review the regulations. A bit surprising that we get yet another request here when everything that is written has been carefully described before.*

[GP]

#### Quotes From Pharmacists

*We have to approve ‘changes’ if the GP has only renewed a prescription for on-demand medicine – this is unnecessary and time-consuming.*

[Pharmacist]

*Want an opportunity to see changes that have been perceived by the system, a report or an overview on the change report when approving the prescription card.*

[Pharmacist]

## Discussion

### Principal Findings

This study explored the challenges associated with the use of eMDD in Norway, focusing on the perspectives of GPs and pharmacists. The findings reveal that while eMDD systems offer significant advantages over traditional paper-based multidose systems—particularly in terms of medication safety and workflow efficiency—they also introduce new complexities. These challenges include issues related to training, system usability, communication, role clarity, and medication safety (Table 1).

The thematic analysis identified 7 key areas of concern: training, system and technology, communication and interaction, division of responsibilities, medication safety, time and resource use, and broader implementation challenges. These findings are consistent with previous research, which has documented similar issues during the transition from paper-based to electronic systems [8-16].

**Table 1.** Summary of findings and improvement proposals.

Theme	Findings	Improvement proposals
Training	Training experiences varied: pharmacists rated it positively, whereas GPs^a^ were more critical.	GPs suggested better access to educational resources, mandatory training, follow-up sessions, and improved user support.
System and technology	Participants reported system slowness, integration errors, and poor usability. Only 11 of 70 experienced no issues.	Suggestions included simplifying processes, improving functionality, and reducing duplicated work.
Communication and interaction	Most participants reported issues, such as increased dialogue messages, misunderstandings between services, and lack of standardization. Thirteen out of 70 did not experience these challenges.	GPs called for clearer responsibilities and better discharge documentation, whereas pharmacists emphasized prioritizing and expediting message handling to avoid delays.
Division of responsibilities	Coordination and role ambiguity were major challenges. Nineteen out of 70 participants reported shifting responsibilities, whereas 18 did not experience this.	Participants called for clearer definitions of responsibilities between hospitals and municipalities, noting that shifting responsibility often leads to forgotten tasks and delays—especially during holidays or with temporary staff.
Medication safety	Medication safety was the most reported concern, especially among GPs. Key issues included updating medication lists, hospital prescriptions, and communication. Only 4 participants reported no safety-related issues.	GPs and pharmacists suggested clearer routines and better coordination for updating medication lists or starting eMDD^b^, as well as locking e-prescriptions to multidose arrangements to prevent duplicate dispensing.
Time and resource use	Increased tasks, dialogue messages, and coordination demands were key challenges. GPs noted higher time/resource use due to more messages, whereas pharmacists pointed to task load and slow responses.	Participants proposed renewing multiple prescriptions at once, enabling pharmacies to message home care services, and improving eMDD features, such as notifications and change reports. A shared communication platform and better training were also recommended.

a GPs: general practitioners.

b eMDD: electronic prescribing for multidose drug dispensing.

### Comparison With Prior Work

The literature review confirmed that many of the challenges identified in this study are not unique. For instance, studies by Jøsendal and Bergmo [8] and Gullslett and Bergmo [9] highlight common barriers to successful eMDD implementation, including increased workloads, insufficient training, and communication breakdowns. Similarly, technical issues, such as system slowness, error messages, and poor integration with electronic health records, have been widely documented [8,14].

Our findings also align with previous research on the importance of medication reconciliation and the risks associated with inconsistent medication lists [12]. A particularly significant safety concern identified in this study is the ability for patients to collect e-prescribed medications, especially controlled substances from multiple pharmacies. This issue aligns with warnings from the Norwegian Board of Health Supervision and findings from other studies [16].

In a broader international context, Scandinavian countries appear to be at the forefront of integrating eMDD into national e-prescription systems. Norway has implemented a fully digital workflow for eMDD, enabling seamless communication between GPs, pharmacies, and home care services [17]. Finland and Denmark have also integrated eMDD into their e-prescription infrastructures, with Finland legislating its use in 2011 and providing publicly funded access to approximately 95% of eligible patients [17]. Sweden, while having the highest per capita use of automated dose dispensing, has faced challenges related to fragmented prescribing responsibilities and reduced frequency of medication reviews [15].

Outside of Scandinavia, countries such as the Netherlands, Australia, and South Africa have adopted centralized MDD systems, often through hub-and-spoke models [18]. In the Netherlands, Pharmacy Voorzorg serves over 145,000 patients via 540 community pharmacies [18]. South Africa’s semiautomated central dispensing unit supports chronic disease management, including HIV care [18]. It is important to distinguish between eMDD and MDD. While MDD refers to a physical dispensing system or automated dispensing device, eMDD denotes a national e-prescribing system. These systems serve distinct functions within the medication management process.

Despite these advancements, evidence on clinical outcomes and cost-effectiveness remains limited, and concerns persist regarding system usability, role clarity, and medication safety—issues that mirror those identified in this study.

### Implications for Practice

The results highlight the need for a structured, interdisciplinary approach to training. In the first survey question, we asked respondents which areas they found challenging and how frequently these challenges occurred. Many GPs indicated that they did not feel fully prepared, often relying on limited documentation or informal, peer-led guidance. To address these gaps, participants recommended implementing modular e-learning programs, video tutorials, and mandatory onboarding sessions for all users. Pharmacists emphasized the value of practical, hands-on training and expressed a strong interest in gaining deeper insights into the GP workflow to enhance collaboration.

System usability remains a major barrier. Participants described the eMDD interface as unintuitive and cumbersome, citing issues such as excessive clicking, unclear feedback, and time-consuming processes. To improve the user experience, they proposed simplifying workflows, improving system responsiveness, and introducing features such as batch prescription renewal and more transparent error messaging.

Communication between health care providers emerged as another critical issue. Although dialogue messages are intended to facilitate coordination, they often lead to information overload for GPs and delays for pharmacists. Participants recommended the development of a unified communication platform that integrates GPs, pharmacists, and home care services, along with the establishment of clearer protocols for handling medication changes during hospital discharges.

Role ambiguity and inconsistent handovers of responsibility, especially during staff absences or transitions, were also highlighted. Participants called for clearer definitions of responsibilities, particularly regarding hospital physicians’ roles in updating medication lists. The upcoming national rollout of the Patient’s Medication List [19] may help address some of these concerns, but its success will depend on effective implementation and active engagement from all stakeholders.

### Limitations

This study has several limitations that should be acknowledged. The sample size (n=70) limits the generalizability of the findings, and the response rate was affected by undelivered emails and potential selection bias. Participants with strong opinions, either positive or negative, about eMDD may have been more likely to respond, potentially skewing the results. Additionally, the survey did not capture the duration of eMDD use, making it difficult to distinguish implementation-related issues from ongoing challenges.

The mixed methods design provided valuable insights, particularly from the qualitative data, which provided a rich exploration of user experiences. However, the limited quantitative data precluded the inferential statistical analysis. Future research with larger, more representative samples and longitudinal designs could provide a more comprehensive understanding of eMDD adoption and its outcomes.

### Recommendations for Future Research

Further research should explore the long-term effects of eMDD on clinical workflows, medication safety, and patient outcomes and evaluate the effectiveness of different training models and system design improvements. As the national rollout of the Patient’s Medication List advances, studies should assess its integration with eMDD and its impact on interprofessional collaboration and medication reconciliation processes.

### Conclusion

This study examined the experiences of Norwegian GPs and pharmacists with the use of eMDD, focusing on key areas such as training, system usability, communication, division of responsibilities, medication safety, and resource use. Using a mixed methods approach that combined a literature review with survey responses from 70 health care professionals, the study identified recurring challenges that hinder the effective implementation and use of eMDD.

The findings indicated that, while eMDD has considerable potential to improve medication safety and streamline workflows, its current effectiveness is limited by gaps in training, technical inefficiencies, fragmented communication, and unclear role definitions. GPs reported insufficient training and support, whereas pharmacists emphasized the need for better coordination and clearer routines. Both groups expressed concerns about system usability, pointing to issues such as slow performance, frequent error messages, and excessive administrative burden.

Communication between health care providers emerged as a critical barrier. Dialogue messages were often perceived as excessive, irrelevant, or delayed, and the absence of a unified communication platform across care levels further complicates coordination, especially during transitions, such as hospital discharges. Similarly, the lack of clarity in the division of responsibilities, particularly regarding medication list updates, contributed to workflow inefficiencies and potential safety risks.

Despite these challenges, the study revealed a strong willingness among health care professionals to improve the system. In contrast to previous research, which has focused primarily on pharmacists, our survey received a notably high number of responses from GPs. Furthermore, participants provided concrete suggestions for improving existing systems, which strengthen the practical relevance of our findings. The proposed solutions included modular e-learning programs, improved system design, clearer communication protocols, and the ability to lock e-prescriptions to prevent duplicate dispensing.

As Norway continues the national rollout of eMDD and prepares for the broader implementation of the Patient’s Medication List, these findings underscore the importance of involving end users in system development and policy planning. Addressing the identified challenges through targeted training, technical enhancements, and interprofessional collaboration will be essential to realizing the full potential of eMDD in improving medication safety and health care efficiency.

## Supplementary material

10.2196/85384 Multimedia Appendix 1 Structured online survey.

## Abbreviations

e-prescribing: electronic prescribing
eMDD: electronic prescribing for multidose drug dispensing
GP: general practitioner
MDD: multidose drug dispensing

## References

[1] Johnsen E, Jøsendal AV, Bergmo TS (2018). E-multidose er bedre for pasientsikkerheten enn dosett og faks [Article in Norwegian]. Sykepleien Forskning.

[2] Om multidose og de faglige rådene [Web page in Norwegian]. Helsedirektoratet.

[3] Statistikk om e-multidose collected from: microsoft power BI [Web page in Norwegian]. Norsk helsenett.

[4] Om e-resept [Web Page in Norwegian]. Norsk Helsenett.

[5] (2025). Nettskjema [Web page in Norwegian].

[6] Norwegian Agency for Shared Services in Education and Research. Sikt.

[7] Braun V, Clarke V (2006). Using thematic analysis in psychology. Qual Res Psychol.

[8] Josendal AV, Bergmo TS (2021). From paper to e-prescribing of multidose drug dispensing: a qualitative study of workflow in a community care setting. Pharmacy (Basel).

[9] Gullslett MK, Strand Bergmo T (2022). Implementation of E-prescription for multidose dispensed drugs: qualitative study of general practitioners’ experiences. JMIR Hum Factors.

[10] Josendal AV, Bergmo TS, Granas AG (2021). Implementation of a shared medication list in primary care—a controlled pre-post study of medication discrepancies. BMC Health Serv Res.

[11] Jøsendal AV, Bergmo TS (2018). Riktigere legemiddellister med multidose i e-resept Ensuring correct medication lists with electronic multidose prescriptions [Article in Norwegian]. Norsk Farmaceutisk Tidsskrift.

[12] Mamen AV (2016). Viktigheten av legemiddelsamstemming for å sikre trygg overgang til elektronisk multidose [Article in Norwegian]. Norsk Farmaceutisk Tidsskrift.

[13] Jøsendal AV, Bergmo TS From paper-based to electronic prescribing of multidose drug dispensing — effects on pharmacy workload. https://www.ecp.ep.liu.se/index.php/shi/article/view/433.

[14] Bergmo TS, Jøsendal AV, Johnsen E Factors easing the transition from paper to electronic prescribing of multidose dispensed drugs (MDD). https://ep.liu.se/ecp/161/001/ecp19161001.pdf.

[15] Jøsendal AV, Bergmo TS, Granås AG, Olsen RM, Sletvold H (2022). Medication Safety in Municipal Health and Care Services.

[16] Ertesvåg M, Tselishcheva EG Elektronisk" legemidler i bruk"-et blikk inn i framtiden evaluering av pilotprosjektet" legemidler i bruk" i" reseptformidleren" inkludert elektronisk multidose i e-resept 2015 electronic ‘medications in use’—a glimpse into the future: evaluation of the pilot project ‘medications in use’ in the prescription intermediary, including electronic multidose in the e-prescription system]. MSc thesis.

[17] Bergmo T, Jøsendal AV, Kolstrup N, Eriksen M, Johnsen E, Johansen M (2016). Multidose i e-resept – Kunnskapsoppsummering: Erfaring med multidose med fokus på de skandinaviske landene [Report in Norwegian]. https://ehealthresearch.no/files/documents/Rapporter/NSE-rapport_2016-04_Multidose-i-e-resept_delrapport-kunnskapsoppsummering.pdf.

[18] Rechel B (2018). Hub-and-spoke dispensing models for community pharmacies in Europe. Eurohealth (Lond).

[19] Direktoratet for e-helse (2020). Pasientens legemiddelliste: Orientering om status og veien fremover [Report in Norwegian]. https://www.helsedirektoratet.no/rapporter/pasientens-legemiddelliste-orientering-om-status-og-veien-fremover/.

