# Supplementary material for: General Practitioners and Pharmacists’ Perspectives on Electronic Prescribing for Multidose Drug Dispensing: Mixed Methods Study
Source: JMIR Med Inform. 2026 May 6;14:e85384. doi: 10.2196/85384 (PMC13148586; doi:10.2196/85384)
Supplement: Multimedia Appendix 1 [file medinform-v14-e85384-s001.docx]

## **Appendix - Survey**

**Introduction**

1. What is your job title?
2. **Challenges with e-prescribing for MDD (eMD)**
3. How often do you experience challenges in these areas when working with eMD?

- Training
- Journal system/eMD system
- Communication and collaboration
- Distribution of responsibilities
- Patient safety
- Increased resource and time usage
- Other

1. Specify which other challenges you have experienced when using eMD.
2. **Training**
3. How has the training in using eMD been for you at your workplace?
4. What do you think could be improved with the training?
5. **System and technology**
6. What challenges have you experienced with the eMD systems you use at your workplace?
7. What do you think could be improved with the eMD system you use?
8. **Communication and interaction**
9. What do you find challenging with communication and collaboration between healthcare services?
10. What do you think can be improved in terms of communication and collaboration?
11. **Division of responsibilities**
12. What do you think has been challenging regarding the distribution of responsibilities related to eMD?
13. What do you think can be improved in terms of the distribution of responsibilities?
14. **Patient Safety**
15. What do you find challenging with patient safety related to eMD?
16. What do you think can be improved with patient safety?
17. **Time and resource use**
18. How has the use of eMD led to increased time and resource usage at your workplace?
19. What do you think can help to streamline tasks related to eMD?
